# Association between Experimental Pain Measurements and the Central Sensitization Inventory in Patients at Least 3 Months after COVID-19 Infection: A Cross-Sectional Pilot Study

**DOI:** 10.3390/jcm12020661

**Published:** 2023-01-13

**Authors:** Lisa Goudman, Ann De Smedt, Stijn Roggeman, César Fernández-de-las-Peñas, Samar M. Hatem, Marc Schiltz, Maxime Billot, Manuel Roulaud, Philippe Rigoard, Maarten Moens

**Affiliations:** 1 STIMULUS Research Group, Vrije Universiteit Brussel, Laarbeeklaan 103, 1090 Brussels, Belgium; 2 Department of Neurosurgery, Universitair Ziekenhuis Brussel, Laarbeeklaan 101, 1090 Brussels, Belgium; 3 Center for Neurosciences (C4N), Vrije Universiteit Brussel, Laarbeeklaan 103, 1090 Brussels, Belgium; 4 Pain in Motion (PAIN) Research Group, Department of Physiotherapy, Human Physiology and Anatomy, Faculty of Physical Education and Physiotherapy, Vrije Universiteit Brussel, Laarbeeklaan 103, 1090 Brussels, Belgium; 5 Research Foundation Flanders (FWO), Egmontstraat 5, 1000 Brussels, Belgium; 6 Department of Physical Medicine and Rehabilitation, Universitair Ziekenhuis Brussel, Laarbeeklaan 101, 1090 Brussels, Belgium; 7 Department of Physical Therapy, Occupational Therapy, Rehabilitation and Physical Medicine, Universidad Rey Juan Carlos, 28922 Alcorcón, Spain; 8 Center for Neuroplasticity and Pain (CNAP), SMI, Department of Health Science and Technology, Faculty of Medicine, Aalborg University, 9220 Aalborg, Denmark; 9 PRISMATICS Lab (Predictive Research in Spine/Neuromodulation Management and Thoracic Innovation/Cardiac Surgery), Poitiers University Hospital, 86021 Poitiers, France; 10 Department of Spine Surgery & Neuromodulation, Poitiers University Hospital, 86021 Poitiers, France; 11 Pprime Institute UPR 3346, CNRS, ISAE-ENSMA, University of Poitiers, 86360 Chasseneuil-du-Poitou, France; 12 Department of Radiology, Universitair Ziekenhuis Brussel, Laarbeeklaan 101, 1090 Brussels, Belgium

**Keywords:** post-COVID-19 condition, persisting symptoms, sensitivity, central sensitization

## Abstract

Fatigue, pain, headache, brain fog, anosmia, ageusia, mood symptoms, and sleep disorders are symptoms commonly experienced by people with post-COVID-19 condition. These symptoms could be considered as manifestations of central sensitization. The aim of this study is to evaluate whether there are indicators of central sensitization by using experimental pain measurements and to determine their association with patient-reported outcome measures (PROMs). A cross-sectional study including 42 patients after COVID-19 infection was conducted. The central sensitization inventory (CSI) was administered as a PROM to evaluate central-sensitization-associated symptoms. Pressure pain thresholds (PPT), temporal summation, and descending nociceptive pain inhibition (CPM) were assessed as experimental pain measurements. The median score on the CSI was 46.5 (Q1–Q3: 33–54). The presence of central-sensitization-associated symptoms was seen in 64.3% of patients based on the CSI (≥40/100 points). A deficient CPM was seen in 12% and 14% of patients when measured at the trapezius and rectus femoris, respectively. A negative correlation between pressure sensitivity on the rectus femoris and the CSI score (r = −0.36, 95%CI −0.13 to −0.65, *p* = 0.007) was observed. Central-sensitization-associated symptoms were present in up to 64.3% of patients post-COVID-19 infection, based on a PROM, i.e., the CSI. A more objective evaluation of nociceptive processing through experimental pain measurements was less suggestive of indicators of central sensitization. Only a small negative correlation between pressure sensitivity and the CSI was observed, thereby pointing towards the discrepancy between the CSI and experimental pain measurements and presumably the complementary need for both to evaluate potential indicators of central sensitization in this population.

## 1. Introduction

The coronavirus disease 2019 (COVID-19) pandemic, caused by severe acute respiratory syndrome coronavirus 2 (SARS-CoV-2) infection, has resulted in worldwide strict lockdowns, physical distancing, and home isolation to slow down the spread of the pandemic and to avoid fatalities [1,2]. Due to the lack of a definitive treatment option for COVID-19, more than 230 vaccine candidates have been proposed, with 49 approved vaccines up until now [3]. A network meta-analysis revealed that COVID-19 vaccines provided a significant reduction in the risk of contracting symptomatic SARS-CoV-2 and a significant reduction in the risk of developing severe COVID-19 in comparison to a placebo [2]. Nevertheless, despite the efficacy of COVID-19 vaccines, the prevalence of persons infected and confronted with persisting symptoms for months or years after SARS-CoV-2 infection is estimated to be up to 31% [4,5]. Fatigue, pain, headache, brain fog, anosmia, ageusia, and emotional or sleep disorders are among the most common persistent post-COVID manifestations [4,6,7,8]. A meta-analysis reported that fatigue is the most prevalent post-COVID symptom with a prevalence rate of up to 45% [9]. Patients with these persistent debilitating symptoms are classified as suffering from post-COVID-19 condition, defined as “a condition that occurs in people who have a history of probable or confirmed SARS-CoV-2 infection; usually within three months from the onset of COVID-19, with symptoms and effects that last for at least two months” [10,11]. It is specifically denoted by the WHO that the symptoms of post-COVID-19 condition cannot be explained by an alternative medical diagnosis [10,11].

Fatigue is one of the core symptoms in central-sensitization-associated disorders [12,13], leading to the hypothesis that central sensitization might be an underlying common etiology in chronic pain patients and in patients with post-COVID-19 condition [14]. Post-COVID-19 condition has previously been described as having considerable overlapping symptoms with myalgic encephalomyelitis/chronic fatigue syndrome [15,16], a condition associated with central sensitization [17]. Emerging evidence suggests the presence of central sensitization in a subgroup of patients with post-COVID-19 condition. By using the self-rated questionnaire, the central sensitization inventory (CSI), a Belgian study showed that 70% of individuals with post-COVID-19 condition exhibited sensitization-associated symptomatology [18] whereas a Spanish study reported a prevalence of only 34% in a group of patients exhibiting post-COVID pain [19]. Another rational supporting the presence of central sensitization is the fact that individuals with post-COVID-19 condition exhibit several central nervous-system-derived symptoms, e.g., fatigue, sleep problems, memory loss, concentration problems, or psychological disturbances [4].

The exclusive use of a screening questionnaire such as the CSI for inferring sensitization in people with post-COVID-19 condition is not recommended because a self-reported tool cannot capture the complexity of a nervous system impairment such as central sensitization [20]. Accordingly, besides patient-reported outcome measure (PROMs), i.e., CSI, to identify the presence of central-sensitization-associated symptoms, quantitative sensory testing could be used to quantify sensory dysfunctions by evaluating pain thresholds, nociceptive pain facilitation, and endogenous pain inhibition [21,22]. A less efficacious descending nociceptive inhibitory system, combined with increased nociceptive facilitation and decreased pain thresholds, is commonly related to an increased excitability of the central nervous system, namely central sensitization [23,24]. To the best of our knowledge, no study has previously investigated the presence of altered nociceptive processing by conducting experimental pain measurements in individuals post-COVID-19 infection. Therefore, the aims of this study were to further evaluate whether there are indicators of central sensitization in patients with post-COVID-19 infection by using experimental pain measurements and to determine their association with PROMs such as the CSI.

## 2. Materials and Methods

### 2.1. Study Participants

This is a cross-sectional study investigating the symptoms of central sensitization and impaired nociceptive processing in individuals post-COVID-19 infection. Both male and female patients who had been previously infected with SARS-CoV-2 were eligible to participate. The patients were only eligible if they had had a positive COVID-19 test at least 3 months before inclusion. The patients were recruited through various ways. Firstly, the patients were invited to participate by physicians in cases where they were consulted after a SARS-CoV-2 infection at the Department of Physical Medicine and Rehabilitation of UZ Brussel. Secondly, advertisements and announcements from patient support groups were used as an additional recruitment strategy. Finally, social media was used to recruit patients. The study protocol was approved by the central ethics committee of Universitair Ziekenhuis Brussels (B.U.N. 1432020000348) on 16 December 2020. The study was registered on clinicaltrials.gov (NCT04703452) and was conducted according to the revised Declaration of Helsinki (1998).

### 2.2. Study Protocol

The patients included had one study visit to UZ Brussel, which was scheduled according to patient preferences. During the study visit, the patients first filled out the following three PROMs in a randomized order: the central sensitization inventory, the post-COVID-19 functional status scale, and the London chest activity of daily living. After filling out the questionnaires, the following experimental pain measurements were performed: pressure pain thresholds (PPT), temporal summation, and descending nociceptive inhibition. The complete testing protocol lasted for maximally 1 h. All of the participants were asked to refrain from consuming caffeine, alcohol, or nicotine 24 h before the study.

### 2.3. Self-Reported Questionnaires

The primary outcome was the presence of central-sensitization-associated symptoms, as assessed by the central sensitization inventory (CSI). The CSI consists of 25 symptom-related items that the patient has to score on a five-point Likert scale [25]. A total score of ≥40/100 is indicative of the presence of central sensitization symptomatology (sensitivity: 81% and specificity: 75%) [25]. The participants were categorized based on central-sensitization-related severity into three subgroups: (i) low level, (ii) medium level, or (iii) high level of central-sensitization-related symptom severity using an accessible calculator (https://www.pridedallas.com/questionnaires, accessed on 19 November 2021) [26]. The CSI has good psychometric properties for assessing symptoms of central sensitization [21] and it is validated in Dutch and French [27,28].

Post-COVID-19 functional status was evaluated by the post-COVID-19 functional status scale (PCFS). This scale is ordinal, has six steps ranging from 0 (no symptoms) to 5 (death) and covers the entire range of functional outcomes by focusing on limitations in usual activities either at home or at work/study, as well as lifestyle changes. More specifically, the following scale grades are included: 0 (no functional limitations), 1 (negligible functional limitation), 2 (slight functional limitation), 3 (moderate functional limitation), 4 (severe functional limitation), and death. The PCFS was assessed twice, once by the interviewers during a short-structured interview, as well as with self-reporting by the patient [29,30].

The London chest activity of daily living (LCADL) scale was assessed to evaluate the level of dyspnea during activities of daily living (0–75 points). This questionnaire consists of 15 items and contains the following answer options: 0 (I do not perform this activity because I’ve never had to do it or it is irrelevant), 1 (I do not feel any breathless when performing this activity), 2 (I feel moderate breathless when performing this activity), 3 (I feel a lot of breathless in performing this activity), 4 (I cannot perform this activity due to breathless and I have no one who can perform the activity for me) or 5 (I cannot perform this activity anymore and I need someone to perform it for me or help me because of breathless). Greater dyspnea-related limitation in activities of daily living translates into higher scores on the LCADL. It has four domains: self-care (0–20 points), domestic activities (0–30 points), physical activities (0–8 points), and leisure time (0–12 points) [31,32].

### 2.4. Experimental Pain Measurements

Pressure pain sensitivity was measured at the middle of the trapezius muscle (i.e., midway between the spinous process of the seventh cervical vertebra and the lateral edge of the acromion) [33] and at the center of the rectus femoris muscle (i.e., the middle of the distance between the anterior inferior iliac spine and the upper edge of the patella) [34] on the dominant side with a hand-held manual algometer (Wagner FPX™ Algometer, Wagner Instruments, Greenwich, CT, USA). On each location, two measurements (interval 30 s) were obtained, generating a mean pressure pain threshold (PPT) per area that was used in subsequent analyses. To determine the PPT, pressure was increased at a rate of approximately 1 kg/s until the participants indicated that the sensation became painful. Consequently, the pressure was immediately released. The pressure established at that moment was used as PPT, measured in kg/cm^2^. Pressure algometry has been found to be an efficient and reliable technique for PPT determination and examination of sensitivity to pressure pain [35].

Temporal summation (TS) was evaluated with 10 consecutive pressure pulses at the intensity of the PPT (previously determined) on the same areas. For each pulse of the TS procedure, the pressure was increased at a rate of 2 kg/s until the previously determined PPT, where it was maintained for one second before being released. An inter-stimulus interval of one second was used. The participants were instructed to rate the pain intensity of the first, fifth, and tenth pressure pulse according to a verbal numeric pain rating scale (vNPRS). The TS score was obtained by subtracting the first vNPRS score from the last vNPRS [36]. The higher the TS score, the more efficient the nociceptive signaling to the brain. The TS procedure is found to be reliable and valid and is supported for use in chronic pain patients [37].

To assess the efficacy of endogenous pain inhibition, the conditioned pain modulation (CPM) paradigm was used [38,39,40]. An occlusion cuff was used as a conditioning stimulus and pressure stimuli were used as the test stimulus on the trapezius and quadriceps locations. The conditioning stimulus was applied to the non-dominant side [34] for 2 min. In order to apply the conditioning stimulus, an occlusion cuff was inflated to a painful intensity and maintained at that level as a heterotopic noxious conditioning stimulus. The cuff was inflated at approximately 20 mmHg/s until the point that the sensation first became painful. Next, patients adapted for 30 s to the stimulus and subsequently rated their pain intensity on the vNPRS. Cuff inflation was then increased or decreased until the participant indicated that the pain level was equal to a score of 3/10 on the vNPRS. Thirty seconds after application of the conditioning stimulus, the first test stimulus (via PPT) was measured on the trapezius. After 60 s, the second test stimulus on the trapezius was assessed. Again, 30 s later (at 1′30″ after application of the conditioning stimulus), the first test stimulus on the quadriceps muscle was applied (via PPT) and the last test stimulus on the quadriceps was applied again 30 s later (at 2′) [34]. The CPM effect is calculated as PPT after conditioning stimulation—PPT before conditioning stimulation [41]. Negative scores (decreased PPTs) indicate no CPM effect and positive scores (increased PPTs) indicate effective CPM.

### 2.5. Statistical Analysis

All of the analyses were performed in R Studio version 1.4.1106 (R version 4.0.5, R Foundation). *p*-values of 0.05 or less were considered statistically significant. Statistics for quantitative variables included median and 1st and 3rd quartiles, the number of observations, and number of missing values. For categorical variables, the absolute counts (*n*) and percentages (%) of patients were presented.

The primary outcome of this study is to identify whether patients post-COVID-19 infection suffer from central-sensitization-associated symptoms, based on the total CSI score. Therefore, the percentages of patients with central sensitization were reported based on the cut-off value of 40/100 on the CSI and the central-sensitization-related symptom severity categories. Furthermore, correlation analyses were performed between total CSI scores and experimental pain measurements (i.e., PPT, TS, and CPM) and the total LCADL score using Spearman rank correlations. A Bonferroni correction was applied to correct for multiple testing (seven comparisons for each variable). Agreement between the PCFS scores rated by the patient and the physician was evaluated using a weighted kappa with linear weights. Kruskal–Wallis tests were used to explore the effect of the presence of symptoms of central sensitization on experimental pain measurements, total LCADL scores, and PCFS scores.

Finally, K-means clustering was performed to classify patients according to shared features of self-reported data and experimental measurements. For the clustering, the centroids of hierarchical clustering (squared Euclidean distances with the method of Ward) were used as starting points. All of the variables were standardized before clustering and only patients with complete data were incorporated into the cluster analysis. All of the analyses were performed on the data as observed. This means that no data imputation strategies, nor observation carried forward techniques, were performed.

## 3. Results

### 3.1. Demographic Statistics

In total, 42 patients were included in this study (12 males and 30 females) with a mean age of 48 (SD: 12) years. All of the visits took place between 19 January 2021 and 20 December 2021, whereby only one patient was hospitalized for COVID-19 infection at the time of the study visit. The median time between confirmation of SARS-CoV-2 infection and the study visit was 190.5 (Q1–Q3: 117–360) days. Four patients were on non-steroidal anti-inflammatory drugs, four patients were on paracetamol, five patients were on antidepressants, one patient was on opioids, two patients were on benzodiazepines, and one patient received a combination product. For female patients, oral anticonceptives were taken by two patients and two patients took hormone replacement therapy.

### 3.2. Symptoms of Central Sensitization, Functionality, and Disability

The median score on the CSI was 46.5 points (Q1–Q3: 33–54), whereby 15 patients (35.7%) had a score < 40/100 on the CSI and 27 patients (64.3%) had a score ≥ 40/100. In terms of severity, 5 patients (11.9%) could be classified as low level of central-sensitization-related symptom severity (CSI median score: 18 points, Q1–Q3: 8–21), 12 (28.6%) as medium-level (CSI median score: 33 points, Q1–Q3: 29.75–36.25), and 25 (59.5%) as high-level of central-sensitization-related symptom severity (CSI median score 53 points, Q1–Q3: 48–60). The median CSI score in males (CSI median score: 33.5 points, Q1–Q3: 26.25–46.5) was significantly lower compared to females (CSI median score: 49.5 points, Q1–Q3: 37.75–58) (sample difference −13 (95% CI −2 to −24), *p* = 0.02).

Concerning the PCFS, 4 persons (9.5%) had no functional limitations (grade 0), 7 (16.7%) had negligible functional limitations (grade 1), 15 (35.7%) reported slight functional limitations (grade 2), 14 (33.3%) indicated moderate functional limitations (grade 3), and 2 (4.8%) scored severe functional limitations (grade 4) according to the patient ratings. For the interviews conducted by the physician, 2 persons (4.8%) had no functional limitations (grade 0), 6 (14.3%) had negligible functional limitations (grade 1), 14 (33.3%) reported slight functional limitations (grade 2), 18 (42.8%) indicated moderate functional limitations (grade 3), and 1 (2.4%) scored severe functional limitations (grade 4). There was missing data for one patient (2.4%). The median time to conduct this interview entailed 1245 (Q1–Q3: 738–2218) seconds, corresponding to 20.75 min. There was a statistically significant agreement between both ratings, kw = 0.546 (95% CI 0.345 to 0.748, *p* < 0.001). The strength of agreement was classified as moderate [42]. No statistically significant differences were revealed for PCFS scores between the males and females, based on patient ratings (W = 136.5, *p* = 0.21) and interviews (W = 160.5, *p* = 0.90). Table 1 provides an overview of the classifications based on self-reports and interviews.

Concerning the LCADL, median scores of 22/75 (Q1–Q3: 17.25–27.50) were revealed, whereby higher scores indicate higher dyspnea-related limitation in activities of daily living. For self-care, a median value of 4.5/20 (Q1–Q3: 4–6) was revealed. For domestic activities, physical activities and leisure time median values of 8.5/30 (Q1–Q3: 6–11.75), 4/8 (Q1–Q3: 3–4) and 4/12 (Q1–Q3: 3–5) were reported, respectively. The median LCADL score in males (LCADL median score: 16 points, Q1–Q3: 15–22.75) was significantly lower compared to females (LCADL median score: 23.5 points, Q1–Q3: 20–30.25) (sample difference −6 (95% CI −1 to −10), *p* = 0.02).

### 3.3. Experimental Pain Measurements

The median PPT on the trapezius muscle was 4.4 (Q1–Q3: 3.1–5.6) kg/cm^2^ and 4.4 (Q1–Q3: 3.4–6.5) kg/cm^2^ on the quadriceps muscle. For temporal summation, the first score was subtracted from the final score, leading to a median value of 3 (Q1–Q3: 1.25–4) for the trapezius and 4 (Q1–Q3: 2–5) for the quadriceps point.

For functioning of the descending inhibitory pathways, 35 patients demonstrated an efficient CPM (median CPM effect: 1.28, Q1–Q3: 0.73 to 1.79) and 5 patients did not (median CPM effect: −0.50, Q1–Q3: −0.82 to −0.22) when measured on the trapezius muscle. On the quadriceps muscle, 6 patients did not demonstrate an efficient CPM (median CPM effect: −0.01, Q1–Q3: −0.04 to 0.0), while 34 patients demonstrated an efficient CPM (median CPM effect: 1.53, Q1–Q3: 0.85 to 2.32). In two patients, no CPM effect was measured.

### 3.4. Associations between Self-Reported and Experimental Measurements

Table 2 presents outcome measurements by severity of central-sensitization-associated symptoms.

**Figure 1:**
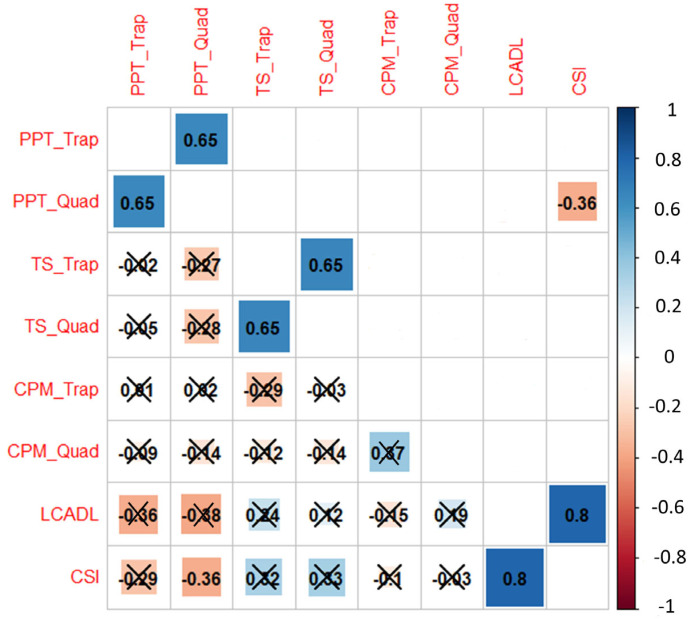
Correlation plot. The correlation coefficients range from −1 (red) to +1 (blue) and are presented with their actual values on the plot. In the lower triangle, correlations that are not statistically significant are marked with a cross. On the upper triangle, only statistically significant correlations are presented. Abbreviations: CPM: conditioned pain modulation, CSI: central sensitization inventory, LCADL: London chest activity of daily living, PFCS: post-COVID-19 functional status scale, PPT: pressure pain threshold, Quad: quadriceps, Trap: trapezius, and TS: temporal summation.

A significant positive correlation between PPTs on the trapezius and quadriceps (r = 0.65, 95% CI 0.54 to 0.85, *p* < 0.001) and between TS on the trapezius and quadriceps (r = 0.65, 95% CI 0.44 to 0.8, *p* < 0.001) was observed. The total scores on the LCADL and CSI were also positively correlated (r = 0.8, 95% CI 0.54 to 0.85, *p* < 0.001). A negative correlation between PPTs on the quadriceps and the CSI score (r = −0.36, 95% CI −0.13 to −0.65, *p* = 0.0067) was also identified (Figure 1).

Based on Kruskal–Wallis tests, there was a significant effect of central sensitization-associated symptoms on PPT on the trapezius at the 5% level (χ² (df = 1) = 4.46, *p* = 0.03) (Figure S1). The effect of the presence of symptoms of central sensitization did not result in statistically significant differences for the other experimental pain measurements.

For the LCADL, the median score in patients with the presence of symptoms of central sensitization was higher compared to patients with the absence of these symptoms (χ² (df = 1) = 27.18, *p* < 0.001). There was a significant effect of central sensitization-associated symptoms on the PCFS with higher median values in patients with sensitization symptomatology, both for the PCFS scores derived from the self-reporting of patients (χ² (df = 1) = 12.67, *p* < 0.001) as well as from the interviews (χ² (df = 1) = 9.91, *p* = 0.002, Figure S2).

**Figure 2:**
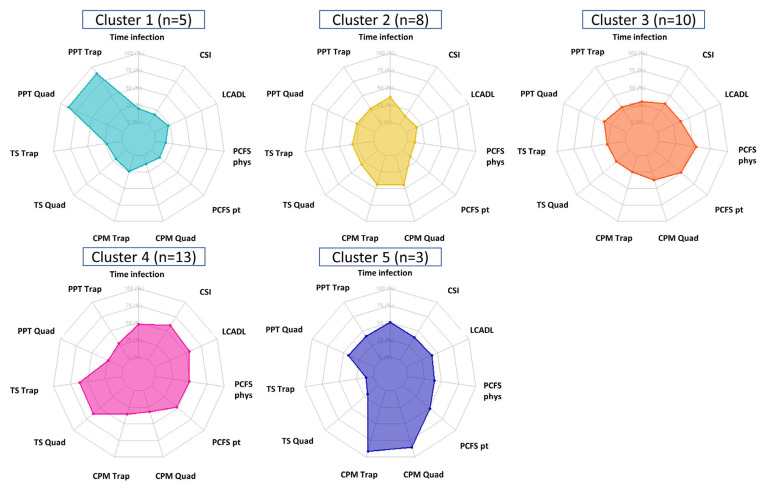
K-means clustering algorithm with five distinct clusters. CPM was coded negatively in case there was a malfunctioning of the descending nociceptive inhibitory pathways and positive in case of functioning of these pathways. For temporal summation, higher scores indicate more nociceptive facilitation. Abbreviations. CPM: conditioned pain modulation, CSI: central sensitization inventory, LCADL: London chest activity of daily living, PFCS: post-COVID-19 functional status scale, phys: physician, PPT: pressure pain threshold, pt: patient, Quad: quadriceps, Trap: trapezius, and TS: temporal summation.

The K-means clustering resulted in five clusters, whereby 5, 8, 10, 13, and 3 patients were classified (*n* = 39). A visual presentation of the clusters is graphically presented in Figure 2.

Cluster 4 represented 33.3% of the patients and could be interpreted as a typical chronic pain patient showing a relatively high CSI score, nociceptive facilitation, malfunctioning of the nociceptive inhibitory pathways, and lower PPTs as compared to the remaining clusters. Cluster 1 (12.8%) is characterized by high PPTs on the trapezius and rectus femoris, while cluster 5 (7.7%) is characterized by high values of descending nociceptive inhibitory control. Cluster 3 (25.6%) predominantly reveals a high contribution of limitations in usual activities based on the PCFS. Finally, 20.5% of patients are attributed to cluster 2 in which rather low PPTs, lower contribution of PROMs, high values of descending inhibitory control and a limited contribution of nociceptive facilitation are present.

## 4. Discussion

The current study observed the presence of central-sensitization-associated symptomatology in up to 64.3% of patients post-COVID-19 infection, based on self-reporting through the CSI. Most patients (69%) reported slight to moderate functional limitations. Physical activities were responsible for most of the dyspnea-related limitations in activities of daily life. For patients with central-sensitization-associated symptoms, greater dyspnea-related limitations and higher limitations in functional status were observed. A small negative correlation between pressure pain sensitivity on the rectus femoris and the CSI score was observed. No other correlations between experimental pain measurements and CSI were observed. Clustering analysis identified that 33% of patients in this sample demonstrated a profile featuring a high CSI score, nociceptive pain facilitation, and malfunctioning of the nociceptive inhibitory pathways.

Based on the WHO definition, individuals with a history of probable or confirmed SARS-CoV-2 infection, usually 3 months from the onset of COVID-19 with symptoms that last for at least 2 months, could be classified as suffering from post-COVID-19 condition [10]. The patients included in this study had a positive COVID-19 test at least 3 months before study inclusion, nevertheless the exact duration of symptomatology was not asked. Only 9.5% of patients did not report any symptoms of functional limitations, therefore it is assumed that more than 90% of patients suffered from post-COVID-19 condition. We identified the presence of central-sensitization-associated symptomatology in up to 64.3% of our patients post-COVID-19 infection based on PROMs. This result is similar to our previous study [18], but much higher than the prevalence rate of 34% observed in individuals with just post-COVID pain [19]. This discrepancy may be related to the fact that the sample of patients included by Fernández-de-las-Peñas et al. mainly suffered from solely post-COVID pain [19] whereas the Belgian sample [18] reported more heterogeneous post-COVID symptoms such as fatigue or memory loss, which are also evaluated in the CSI [25]. In fact, the presence of psychological and physical post-COVID symptoms are related to the CSI score. Interestingly, higher CSI scores were associated with greater dyspnea-related limitations and higher functional limitations in the current study, thus supporting the previous assumption.

Since the use of PROMs such as the CSI is not able to further ascertain the presence of altered nociceptive pain processing, we included, for the first time, experimental pain measurements in people post-COVID-19 infection. The presence of pressure pain hyperalgesia increased temporal summation and impaired descending inhibition are manifestations of central nervous system sensitization [43]. We were unable to determine the presence of pressure hyperalgesia in our sample of individuals post-COVID-19 infection since we did not include a control group without post-COVID symptoms; however, it would be expected for lower PPTs in individuals with persistent symptoms to be found. Historical data are available for pain-free populations with testing on the trapezius whereby mean values ranging from 4.96 (SD: 3.33) [44] to 5.32 (SD: 3.28) [44], and 5.75 (SD: 2.88) [45] were revealed. Mean PPT values of 4.02 (SD: 1.60) [45] have been observed in patients with whiplash-associated disorders and 2.90 (SD: 2.49) [44] in patients with fibromyalgia. The currently obtained values in patients post-COVID-19 infection seem to be in line with the findings observed in patients with whiplash-associated disorders, suggesting that patients post-COVID-19 infection could also demonstrate pressure pain hyperalgesia.

Nevertheless, our main objective was to further identify the association of experimental pain measurements with the self-reported CSI. We identified a small correlation between sensitivity to pressure pain and the CSI score suggesting that both outcomes represent different aspects of the sensitization spectrum. This weak correlation is in line with previous data in chronic spinal pain patients where a weak correlation was found between the CSI and PPT [46], as was the case in patients with knee osteoarthritis [47]. Statistically, non-significant results were revealed between the CSI and PPT in patients with shoulder pain [48] and patients with chronic whiplash-associated disorders [49]. No other association between the CSI with temporal summation or CPM was identified in the present study, as was the case in patients with chronic whiplash-associated disorders [49]. These results further support the belief that the CSI assessed a broad spectrum of sensitization symptomatology and not just altered nociceptive pain processing. As such, the CSI only marginally reflects direct alterations in central nociceptive processing and is better at identifying psychosocial factors that patients experience than identifying central nervous system adaptations due to central sensitization [49]. It is also possible that the small percentage of patients showing an impaired CPM explains the lack of association.

Concerning the PROMs, a positive correlation was revealed between the LCADL and the CSI (r = 0.8, 95% CI 0.54 to 0.85, *p* < 0.001), meaning that patients with higher symptomatology of central sensitization present more dyspnea during activities of daily living. Furthermore, patients with high central sensitization symptomatology also revealed more limitations in functional outcomes. Previous studies have already demonstrated correlations between CSI and functionality, work ability, depression, and social support scales in patients with spinal pain [50,51,52]. These findings further support the previous hypothesis that the CSI captures a broad spectrum of symptomatology and not only evaluates central nervous system processing in response to nociception. The currently obtained values on the LCADL (median 22/75, Q1–Q3: 17.25–27.50) for dyspnea-related limitation in activities of daily living are in line with the mean value of 17 (SD: 5.7) that was revealed in a population of post-COVID-19 patients in Turkey [53]. Despite the fact that most patients in this study were female (71.4%), females had more dyspnea-related limitations in activities of daily living and more central-sensitization-associated symptoms compared to males. A previous study explored phenotypes based on clinical data obtained in a post-COVID-19 care clinic and revealed that the fatigue-predominant phenotype was more common in females, while the dyspnea-predominant phenotype was more common in males [54]. Since fatigue is one of the core symptoms in central-sensitization-associated disorders, the results of higher CSI scores in females compared to males is not surprising. Further research is needed to further evaluate the dominance of dyspnea-related limitations in patients post-COVID-19 infection.

Although preliminary due to the low number of participants and no external validation, clustering analysis identified that 33% of patients demonstrated a central sensitization profile featuring a high CSI score, nociceptive pain facilitation, and malfunctioning of the nociceptive inhibitory pathways. This subgroup of patients fulfills all of the criteria for a nociplastic condition [55] as has been proposed recently [56] and this could need particular attention in their management. For instance, early treatment of these subgroups of patients could be applied to avoid the further development of central sensitization. Similarly, more multidisciplinary interventions targeting the nervous system, e.g., acceptance and commitment therapy and pain neuroscience education [57], should be applied to this subgroup of patients. These hypotheses should be confirmed or refuted in future clinical trials. Additionally, further exploration and validation of the clustering analysis should still be performed, incorporating the clinical features of pain.

Despite the innovative aspect of conducting experimental pain measurements in patients post-COVID-19 infection, certain limitations should be taken into account when considering the results of this study. The patients included in this study were recruited through convenience sampling, which could limit the generalizability of the results. Nevertheless, the results obtained in this study seem to be comparable to findings in other chronic pain populations. Additionally, no control group was included; therefore, experimental pain measurements could only be compared to data from historical studies. In terms of experimental pain measurements, only one modality (i.e., pressure stimuli) was used, whereas the available guidelines recommend the use of different modalities [39]. This choice was made not to further increase the burden on the study participants, since this was a pilot study exploring indicators of central sensitization in this population. Additionally, standardized sites of stimulation (i.e., trapezius muscle and quadriceps muscle) were used to enable interpretation of the results at the cohort level, regardless of the location of symptomatology of individual patients. Moreover, this study only explored indicators of central sensitization post-COVID-19 infection, without evaluating previous existing comorbidities. Finally, no information was collected on the duration of sick leave or work status after COVID-19 infection.

## 5. Conclusions

In patients post-COVID-19 infection, symptoms of central sensitization were present in 64.3% of the sample based on a self-reported questionnaire. A more objective evaluation of nociceptive pain processing was less suggestive for indicators of central sensitization, thereby pointing towards a discrepancy between the CSI and experimental pain measurements in patients post-COVID-19 infection.

## Figures and Tables

**Table 1:** Agreement and disagreement in PCFS scores between patient self-reporting and physician interviews. Green boxes indicate agreement between both ratings; blue boxes indicate a lower self-reporting score compared to the physician interview; and yellow boxes indicate a higher self-reporting score compared to the physician interview. Abbreviations. PFCS: post-COVID-19 functional status scale.

		Physician PCFS Score					
		0	1	2	3	4	5
**Patient PCFS score**	**0**	2	1	0	0	0	0
	**1**	0	4	2	1	0	0
	**2**	0	1	7	7	0	0
	**3**	0	0	5	9	0	0
	**4**	0	0	0	1	1	0
	**5**	0	0	0	0	0	0

**Table 2:** Total scores on LCADL and PCFS, and results from experimental pain measurements from the complete sample, as well as those separated by the presence or absence (CSI < 40) of symptoms of central sensitization (CSI ≥ 40). Abbreviations: CPM: conditioned pain modulation, CSI: central sensitization inventory, LCADL: London chest activity of daily living, PFCS: post-COVID-19 functional status scale, PPT: pressure pain threshold, and TS: temporal summation.

Variable	Level	Total Sample (*n* = 42)	CSI Score < 40/100 (*n* = 15)	CSI Score ≥ 40/100 (*n* = 27)
Sex	Male/Female	28.6%/71.4%	46.7%/53.3%	18.5%/81.5%
Age (years)		48 (SD: 12)	49 (SD: 15)	48 (SD: 11)
Time since COVID-19 (days)		190.5 (Q1–Q3: 117–360)	184 (Q1–Q3: 96–200)	212 (Q1–Q3: 127–387)
PPT Trapezius		4.37 (Q1–Q3: 3.12–5.60)	5.21 (Q1–Q3: 3.89–7.22)	3.96 (Q1–Q3: 2.82–5.05)
PPT Quadriceps		4.43 (Q1–Q3: 3.40–6.49)	6.21 (Q1–Q3: 3.82–9.13)	4.33 (Q1–Q3: 3.28–5.88)
TS Trapezius		3 (Q1–Q3: 1.25–4)	3 (Q1–Q3: 1.5–3)	4 (Q1–Q3: 1.5–5)
TS Quadriceps		4 (Q1–Q3: 2–5)	3 (Q1–Q3: 2.5–4)	4 (Q1–Q3: 2–5.5)
CPM Trapezius		1.17 (Q1–Q3: 0.53–1.75)	1.36 (Q1–Q3: 0.90–1.75)	0.94 (Q1–Q3: 0.50–1.57)
CPM Quadriceps		1.30 (Q1–Q3: 0.58–2.24)	0.89 (Q1–Q3: 0.47–1.87)	1.52 (Q1–Q3: 0.77–2.31)
LCADL		22/75 (Q1–Q3: 17.25–27.50	15 (Q1–Q3: 15–17.5)	25 (Q1–Q3: 22.5–32.5)
PCFS patient	Grade 0	9.5%	26.7%	0.0%
	Grade 1	16.7%	33.3%	7.4%
	Grade 2	35.7%	26.7%	40.7%
	Grade 3	33.3%	13.3%	44.4%
	Grade 4	4.8%	0.0%	7.4%
PCFS physician	Grade 0	4.8%	13.3%	0.0%
	Grade 1	14.3%	33.3%	3.7%
	Grade 2	33.3%	26.7%	37.0%
	Grade 3	42.8%	20.0%	55.5%
	Grade 4	2.4%	0.0%	3.7%
	Unknown	2.4%	6.7%	0.0%

## Data Availability

The data presented in this study are available on motivated request from the corresponding author.

## Supplementary Materials

The following supporting information can be downloaded at: https://www.mdpi.com/article/10.3390/jcm12020661/s1, Figure S1: Boxplots of the different experimental pain measurements by the presence of symptoms of central sensitization. Figure S2: Boxplots of the LCADL and PCFS scores, separated by the presence of symptoms of central sensitization.
