# Supplementary material for: Association between Experimental Pain Measurements and the Central Sensitization Inventory in Patients at Least 3 Months after COVID-19 Infection: A Cross-Sectional Pilot Study
Source: J Clin Med. 2023 Jan 13;12(2):661. doi: 10.3390/jcm12020661 (PMC9862134; doi:10.3390/jcm12020661)
Supplement: Supplementary file 1 [file jcm-12-00661-s001.zip › jcm-2089484-supplementary.pdf]

**Supplementary Material**

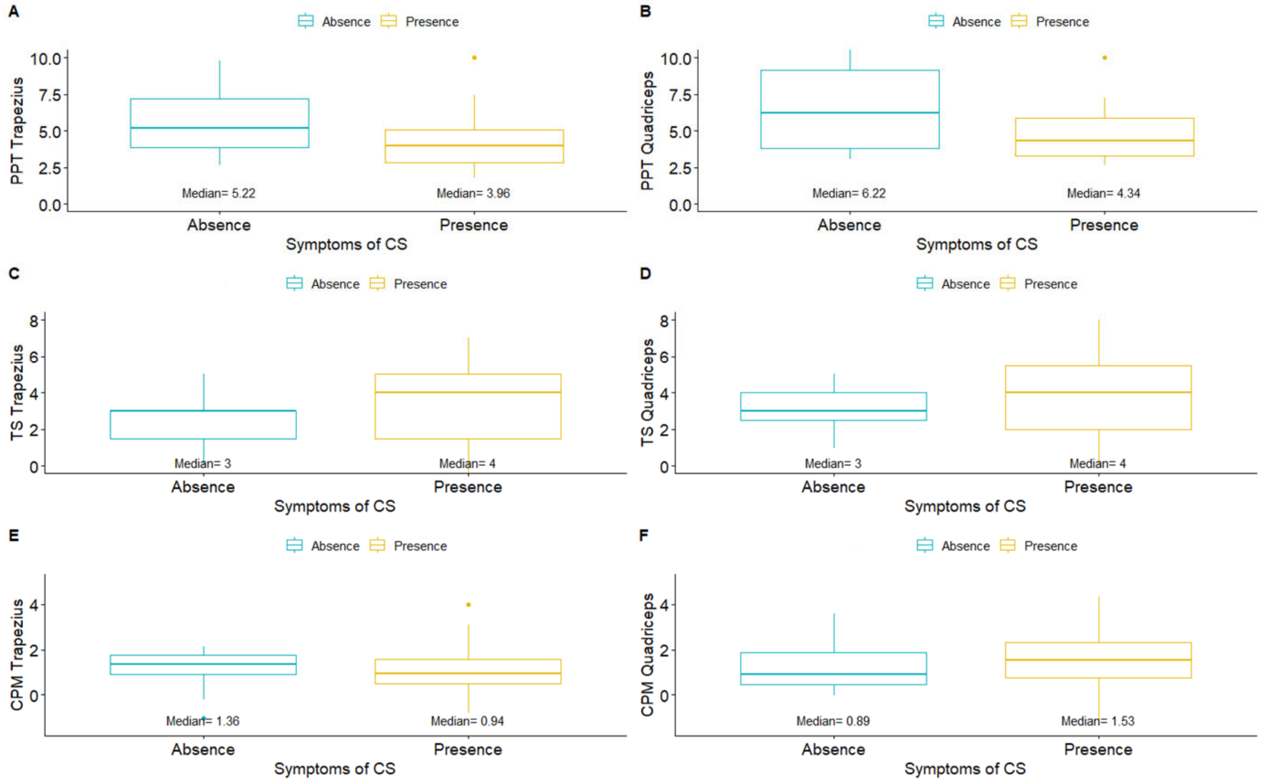


**Figure S1.** Boxplots of the different experimental pain measurements by presence of symptoms of central sensitization. Total scores <40/100 are denoted as absence of symptoms of central sensitization (blue boxes). Total scores ≥40/100 are denoted as presence of symptoms of central sensitization (yellox boxes). Abbreviations. CPM: Conditioned Pain Modulation, CS: Central Sensitization, CSI: Central Sensitization Inventory, PPT: pressure pain threshold, Quad: Quadriceps, Trap: Trapezius, TS: temporal summation.

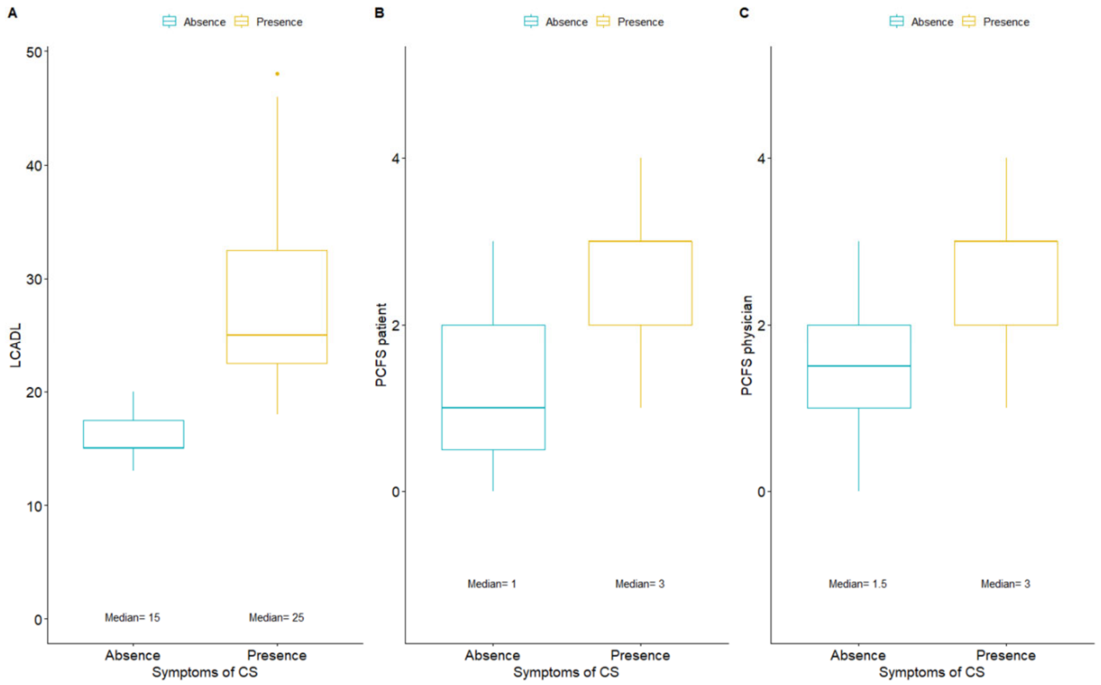


**Figure S2: Boxplots of the LCADL and PCFS scores, separated by presence of symptoms of central sensitization.** Total scores <40/100 are denoted as absence of symptoms of central sensitization. (blue boxes). Total scores ≥40/100 are denoted as presence of symptoms of central sensitization. (yellow boxes). Abbreviations. CS: Central Sensitization, CSI: Central Sensitization Inventory, LCADL: London chest Activity of Daily Living, PFCS: Post-COVID-19 Functional Status Scale.
